# Rapid identification of novel protein families using similarity searches

**DOI:** 10.12688/f1000research.17315.1

**Published:** 2018-12-24

**Authors:** Matt Jeffryes, Alex Bateman

**Affiliations:** 1 European Molecular Biology Laboratory, European Bioinformatics Institute (EMBL-EBI), Hinxton, CB10 1SD, UK

**Keywords:** bioinformatics, protein families, locality sensitive hashing, minhash

## Abstract

Protein family databases are an important tool for biologists trying to dissect the function of proteins. Comparing potential new families to the thousands of existing entries is an important task when operating a protein family database. This comparison helps to understand whether a collection of protein regions forms a novel family or has overlaps with existing families of proteins. In this paper, we describe a method for performing this analysis with an adjustable level of accuracy, depending on the desired speed, enabling interactive comparisons. This method is based upon the MinHash algorithm, which we have further extended to calculate the Jaccard containment rather than the Jaccard index of the original MinHash technique. Testing this method with the Pfam protein family database, we are able to compare potential new families to the over 17,000 existing families in Pfam in less than a second, with little loss in accuracy.

## Introduction

Protein family databases are an important resource for biologists seeking to characterise the function of proteins. The domains, motifs and other features found in a protein form an important organisational structure that can be used to design and interpret experiments on the protein of interest. Operating a protein family database, such as Pfam or InterPro, requires the identification of new families, and the ability to compare them with families already in the database. In this paper, we will describe a computationally efficient method for performing this comparison.

Protein family databases generally describe a particular family using a sequence profile, often in the form of a hidden Markov model (HMM) ^1^. The profile HMM is a representation of the multiple sequence alignment of a number of representatives of a family. The likelihood that a given sequence is a member of a family (that is, it has homology with the other members of the family) is thus estimated by the probability of its alignment to this profile HMM.

**Figure 1.**
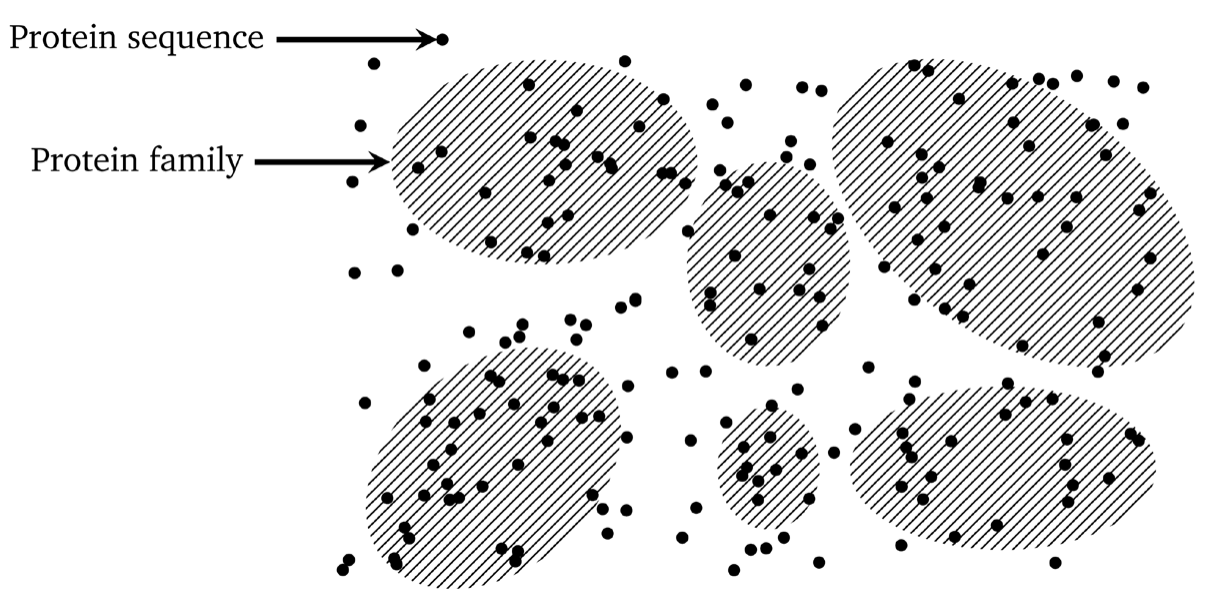
An idealised view of families in sequence space. No sequence is contained in more than one family.

A protein family database should cover as much of sequence space as possible, while reducing overlapping sequence profiles. This is illustrated by the idealised view of sequence space shown in Figure 1.

An overlap occurs when a particular region in a protein sequence is a significant match for more than one sequence profile. In this case there are two possibilities. Either the region of the protein sequence in which the overlapping matches lie is a false positive for one or both of sequence profiles, or the sequence profiles represent families which are homologous, and are in fact a single family. Maximising coverage of sequence space increases the chance of overlap. Each sequence profile added to increase coverage may overlap with the existing profile HMMs in the database. Such overlaps are a result of the fact that HMM sequence profiles are an imperfect model of the underlying homology of the families that they represent.

Pfam is a database of protein families first released in 1997 with models for 175 families. The current release (32.0) contains 17,929 entries ^2, 3^. Each of these entries is described by a seed alignment, which is used to generate a profile HMM, using the HMMER software package. This model is then used to query the protein sequence database UniProtKB, and significant matches for the model are recorded. In Pfam, no region should be matched by more than one model ^4^. Prior to each release of the database, overlap analysis is performed to determine if any residue in UniProtKB is matched by more than one model. This overlap criteria is an important quality control mechanism ^2, 4^. The most recent releases of Pfam have relaxed the overlap criteria slightly, to allow short areas of overlap which do not affect a high proportion of the family members ^3^.

When the authors of Pfam check for overlaps prior to a release, they carry out a complete comparison of all the regions found in every family. This comparison scales with the square of the number of regions in the database. This full comparison is only carried out once per release, and therefore speed is not a significant issue. However, we envision a use case where speed of this overlap test is critical. This use case is the identification of novel families from user sequence similarity searches.

Protein sequence similarity search is used to identify proteins with a similar sequence to a query protein. When two proteins have a similar sequence, it may be inferred that they are evolutionarily related. Thus, the results for a sequence similarity search form a set of potential homologues to a query sequence, which may be thought of as a protein family. The critical question is whether this protein family is novel. If overlaps could be checked quickly enough then the user could be alerted to the fact that the family was novel and be prompted to submit it to Pfam. This overlap check would need to be fast, on the order of a second so that it presented as an interactive element of the search result.

In addition to sequence similarity searches, which find entirely novel groupings of proteins, it can occur that a search matches all members of an existing family along with further proteins which are as yet unclassified in the protein family database. If these proteins are truly homologous with the existing members of the family, then they ought to be members of the family. Therefore, the search may encode a superior model for the existing family.

By analysing the search for overlaps with the families in Pfam, using the method discussed in this paper, searches which could improve Pfam can quickly be identified. This could help curators more rapidly identify novel families, but also open the way for identification of novel user inferred families from sequence search submission.

## Methods

Supposing that we wish to assess a potential new family, for addition to Pfam, we can imagine three scenarios:

1. The search alignment does not overlap with any existing families. It is entirely new residue coverage.
2. The search alignment overlaps with only one existing family.
3. The search alignment overlaps with multiple existing families.

The first case is the clearest case where we may wish to add the family to Pfam. But in the other two cases we may also wish to. In the second case, the search may offer increased residue coverage compared to the existing family. That is, it identifies more members of the family. In the third case, if the multiple families are all of the same Pfam clan, the search may either be a superior model for an existing clan member, or it could be a novel member of the clan. In the second and third case, we require some way of relating an arbitrary search to the families which already exist in Pfam.

### Jaccard index and containment

The Jaccard index of a pair of sets is a measure of their similarity. It is calculated as follows.

$$\;J_{I}(A,B)=\frac{\left|A\cap B\right|}{\left|A\cup B\right|}\;(1)$$

This is the fraction of all members of A and B which are found in both *A* and *B*. Since we are interested in finding search result sets which are supersets of Pfam families, we might also ask what fraction of *A* is found in both *A* and *B*. That is, how close is *A* to being a subset of *B*. This measure is known as containment. We can calculate theb Jaccard containment as follows ^5, 6^.

$$\;J_{C}(A,B)=\frac{\left|A\cap B\right|}{\left|A\right|}\;(2)$$

To calculate either of these statistics requires the use of set intersection between *A* and *B*, which is a computationally relatively expensive operation. Assuming *A* and *B* are stored in hash maps, the average case time complexity is *O*(min( *|A|*, *|B|*)) ^7^.

### Estimation of Jaccard index and containment

Locality sensitive hashing is a technique for quickly identifying similar sets, faster than is possible by calculating the set intersection for each particular pair. Min-wise independent permutations or MinHash is a locality sensitive hash algorithm which estimates the Jaccard index for a pair of sets. Specifically, it estimates the set intersection and union. This additionally allows us to estimate the Jaccard containment. It was was introduced by Broder ^6^, with the original application being the elimination of identical web pages from the index of the Alta Vista search engine.

The algorithm has been applied to a number of biological problems, such as metagenomic clustering, genome assembly, and sequence database search ^8– 12^. MinHash and the Jaccard index and containment for sets *A* and *B* are estimated as follows.

For set *S*, define MIN *_n_*( *S*) as

$$\text{MI}{\text{N}}_{n}(S)=\left\{ \begin{array}{l} \begin{array}{cc} \text{the}\;n\;\text{smallest}\;\text{elements}\;\text{in}\;S & \text{if}|S|\;\geq n; \end{array}\\ \begin{array}{cc} S & \;\text{otherwise}. \end{array} \end{array}\right.\;(3)$$

Let *h*( *x*) be some hash function. Define set *A′* as

A′={h(x)|x∈A}(4)

and *B′* analogously.

We can then see that

$$\text{MI}{\text{N}}_{n}(\text{MI}{\text{N}}_{n}(A\prime )\cup \text{MI}{\text{N}}_{n}(B\prime ))=\text{MI}{\text{N}}_{n}(A\prime \cup B\prime )\;(5)$$

is a sample of at most *n* elements from *A′* ∪ *B′*, and that

$$\text{MI}{\text{N}}_{n}(\text{MI}{\text{N}}_{n}(A\prime )\cup \text{MI}{\text{N}}_{n}(B\prime ))\cap \text{MI}{\text{N}}_{n}(A\prime )\cap \text{MI}{\text{N}}_{n}(B\prime )\;(6)$$

is a sample of at most *n* elements from *A′* ∩ *B′* which are also contained in the sample from *A′* ∪ *B′*. We have ensured that these samples are random by hashing the elements of *A* and *B*. Hence,

$$\frac{\left|\text{MI}{\text{N}}_{n}(\text{MI}{\text{N}}_{n}(A\prime )\cup \text{MI}{\text{N}}_{n}(B\prime ))\cap \text{MI}{\text{N}}_{n}(A\prime )\cap \text{MI}{\text{N}}_{n}(B\prime )\right|}{\left|\text{MI}{\text{N}}_{n}(\text{MI}{\text{N}}_{n}(A\prime )\cup \text{MI}{\text{N}}_{n}(B\prime ))\right|}\;(7)$$

$$=\frac{\left|\text{MI}{\text{N}}_{n}(A\prime \cup B\prime )\cap \text{MI}{\text{N}}_{n}(A\prime )\cap \text{MI}{\text{N}}_{n}(B\prime )\right|}{\left|\text{MI}{\text{N}}_{n}(A\prime \cup B\prime )\right|}\;(8)$$

is an estimate of the Jaccard index. To compute this estimate, only the smallest *n* hashed elements of the sets to be compared is required.

We can take a similar approach for estimating the Jaccard containment. We can find

$$\;\text{MI}{\text{N}}_{n}(A\prime )\cap B\prime \;(9)$$

as elements from *B′* which are also contained in the sample from *A′* and hence

$$\;\frac{\left|\text{MI}{\text{N}}_{n}(A\prime )\cap B\prime \right|}{\left|\text{MI}{\text{N}}_{n}(A\prime )\right|}\;(10)$$

is an estimate of the Jaccard containment.

### Protein family set representation

For Pfam, we wish to determine whether a user’s search overlaps with an existing family. This comparison is on the basis of residues. Hence, the elements of the sets to be compared can be represented uniquely as a combination of a protein’s identifier and residue position within the protein sequence. We can compute the hashes for every family in Pfam. When a user search is performed, we can compute its hash, and estimate whether it falls into one of the desirable categories above (overlapping no families, or covering a single family or clan, with increased residue coverage).

In order to use this method interactively, the time taken to calculate the hash for the user’s search must be taken into account. The hashes for existing Pfam families need only be calculated once, ahead of time, but the hash for a user’s search must be calculated while they are waiting for their search results. For a user search with a large number of results, the number of unique residues within protein sequences which the search aligns to could be in the tens of millions. Each of these residues is an element of the set which must be hashed and sorted in order to produce the hash for the search. The number of set elements can be reduced by sacrificing accuracy. Residues can be grouped together in arbitrary sized chunks. As the size of these chunks increases, the number of elements is reduced, but the risk increases that a search which does not overlap with a Pfam family is misidentified as overlapping with the family. The chunk which a residue with coordinate *i* should be assigned to is computed as ⌊i/w⌋, where *w* is the *window* size of each chunk.

## Results

**Figure 2.**
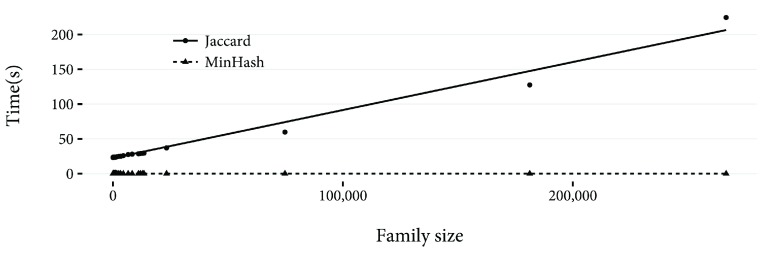
Time taken in seconds to calculate the Jaccard index and to estimate the Jaccard index using MinHash, with *n* = 800, between 50 randomly selected Pfam families and every other family in Pfam. A linear least squares best fit line for the two methods is shown.

**Figure 3.**
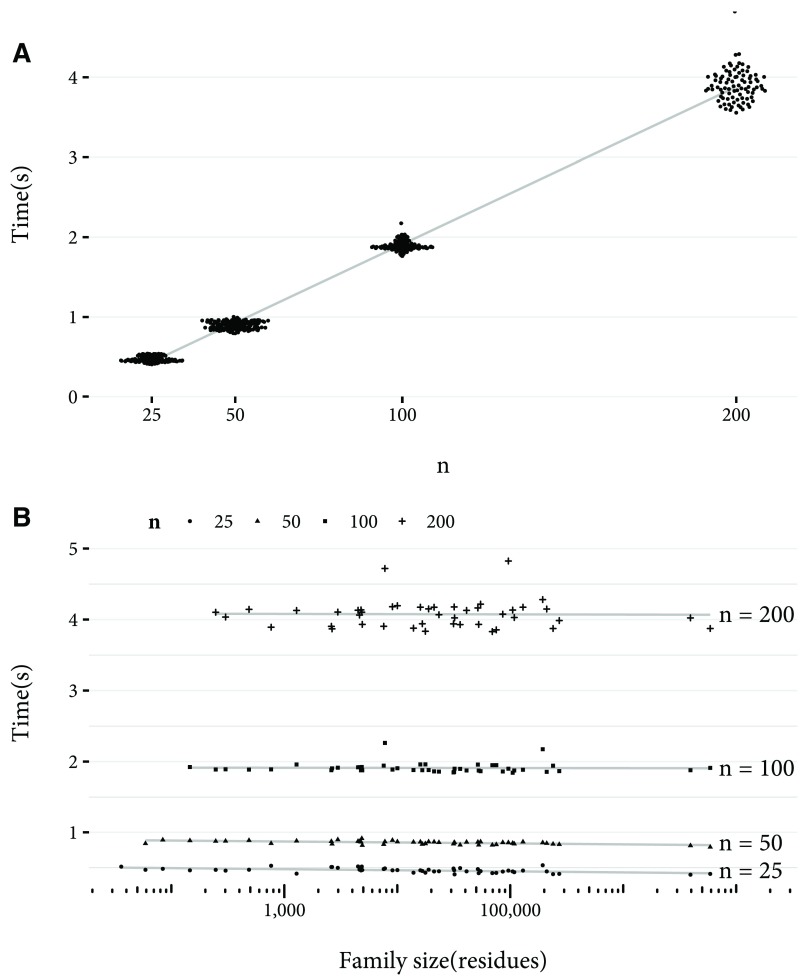
Time to estimate Jaccard index by *n* and family size, for 50 families randomly selected from Pfam 29.0. The time taken for the Jaccard index between the family and the rest of the families to be estimated is shown. For cases where the family size is less than *n*, the results were excluded from the plot. ( **a**) Time taken in seconds to estimate the Jaccard index using MinHash with *n* values of 25, 50, 100, and 200, with *w* value of 1 (that is, no chunking of residues). A linear least squares best fit is shown. Note that the data points are jittered on the *x* axis to better show their distribution. ( **b**) Time taken in seconds to estimate Jaccard index with *n* values of 25, 50, 100, and 200, against family size on a logarithmic scale. A linear least squares best fit for each *n* is shown.

**Figure 4.**
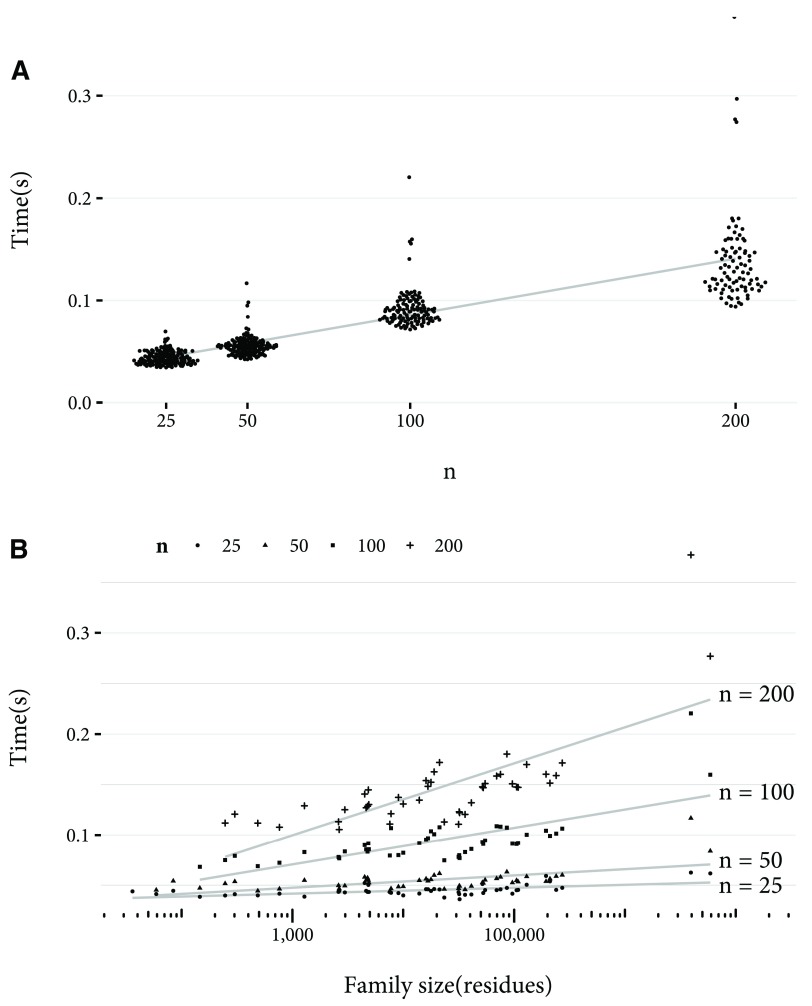
Time to estimate Jaccard containment by *n* and family size, for 50 families randomly selected from Pfam 29.0. The time taken for the Jaccard index between the family and the rest of the families to be estimated is shown. For cases where the family size is less than *n*, the results were excluded from the plot. ( **a**) Time taken in seconds to estimate the Jaccard containment using MinHash with *n* values of 25, 50, 100, and 200, with *w* value of 1 (that is, no chunking of residues). A linear least squares best fit is shown. Note that the data points are jittered on the *x* axis to better show their distribution. ( **b**) Time taken in seconds to estimate Jaccard containment with *n* values of 25, 50, 100, and 200, against family size on a logarithmic scale. A linear least squares best fit for each *n* is shown.

We implemented MinHash in the Python programming language to validate its theoretical gain in performance over exact calculations. We generated hashes of every family in Pfam 29.0 ^3^. We chose 50 random families from Pfam, and for each of these we timed the calculation of the Jaccard index between the family and every family in Pfam, and the MinHash estimate for the Jaccard index with *n* values of 25, 50, 100, and 200. For each method, the calculation was repeated three times, and the minimum of the three used. The results are shown in Figure 2. For any family size, MinHash is faster. Also clear is the linear relationship between family size and calculation time for the Jaccard index. In Figure 3 the linear relationship between n and calculation time for MinHash is shown, and so is the constant time to estimate the Jaccard index as family size varies. In Figure 4, calculation of the Jaccard containment grows with log( *n*). However, for the family sizes tested, calculating the Jaccard containment was faster than the Jaccard index. This is due to the sort operation required to estimate the Jaccard index.

**Figure 5.**
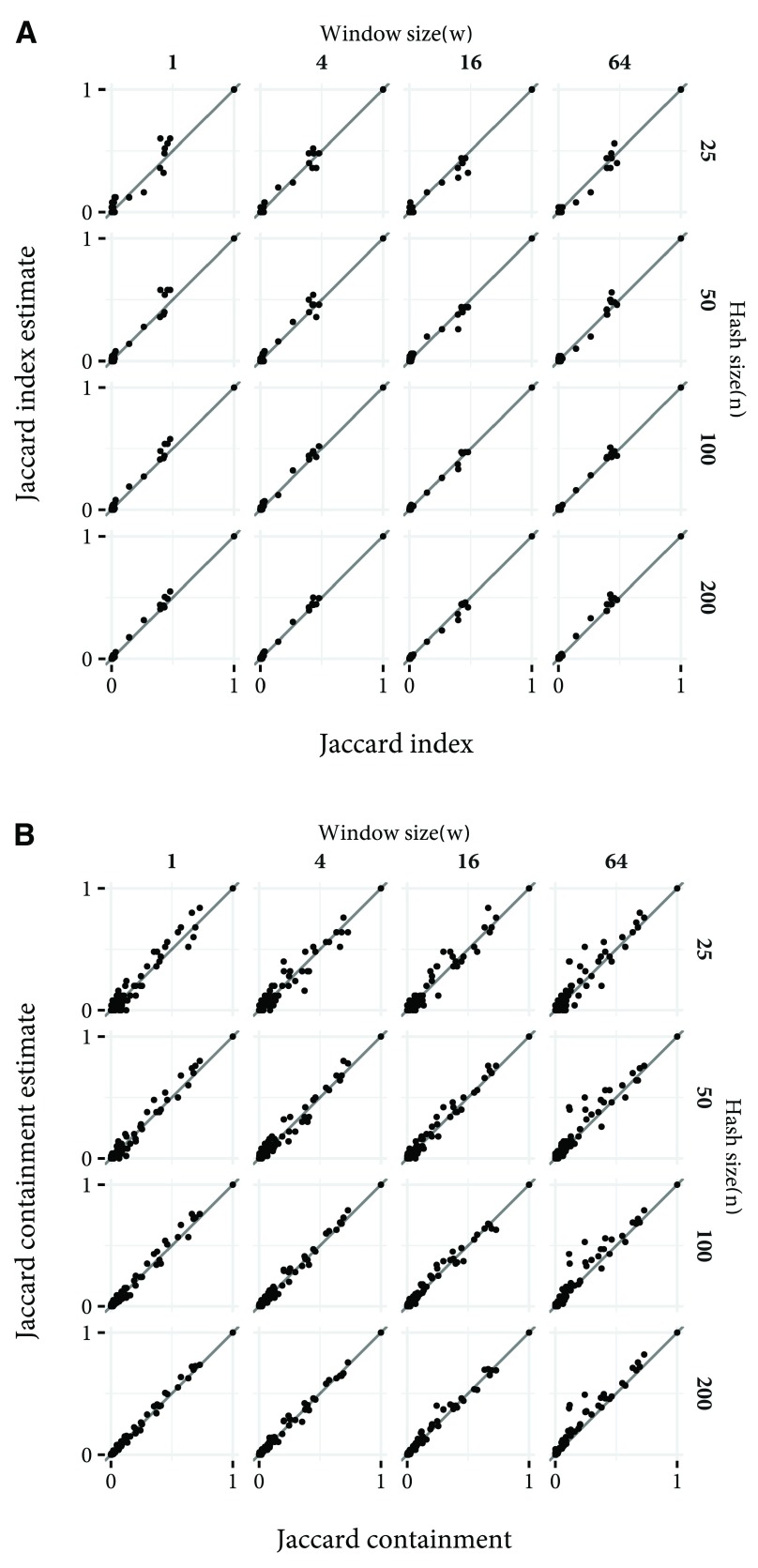
The Jaccard index and containment, and the MinHash estimate of these values between 50 randomly selected Pfam families and every other family in Pfam, for different values of *n* and *w*. The diagonal lines show the position that a perfect estimate would fall. ( **a**) Jaccard index concordance. ( **b**) Jaccard containment concordance.

Calculation time of the order of seconds, even with high values of n, will enable fast estimation of the relationship between a potential new family and the rest of Pfam. In Figure 5, the concordance between Jaccard index and containment and their MinHash estimates, between the same sample as above and the rest of Pfam are shown. Even with *n* = 25 the discrepancy is not great. Thus, searches which may not overlap any existing Pfam family, and families which may be improvements over existing families can be identified in less than a second.

**Figure 6.**
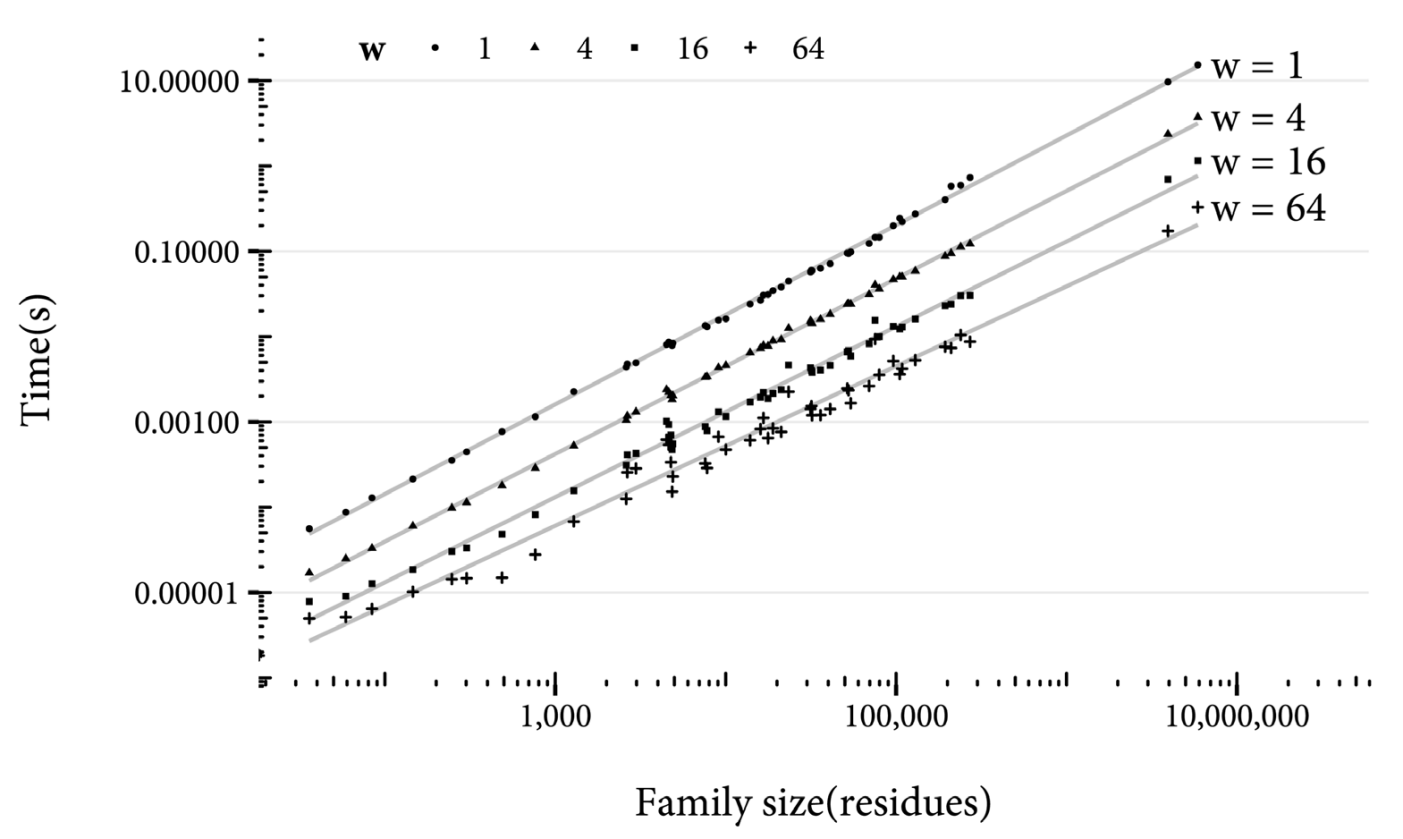
Time taken in seconds to compute the MinHash set hash for against family size, both on a logarithmic scale, for *w* values of 1, 4, 16 and 64.

Increasing the value of *w* also reduces accuracy, but reduces the time to compute the hash of the potential new family. In Figure 6, the time taken to compute hashes for different values of *w* is shown. With a *w* value of 1 (that is, without chunking residues), it takes over 10 seconds to compute the hash of the largest family. Increasing *w* enables this time to be reduced to under a second. For a production system, regular waits of over 10 seconds would be unacceptable, so *w* should be set to at least 4. On the other hand, high values of *w* will result in more frequent errors in multidomain proteins: In cases where the domains have fewer residues separating them than *w* + 1, there is the possibility that the profiles for the two domains could be wrongly identified as overlapping. Therefore, *w* of greater than 16 could be detrimental.

## Conclusions

We have developed a method for quickly comparing the search results produced by querying a profile HMM against a protein sequence database, to a protein family database. We have adapted a method for estimating the Jaccard index of a pair of sets to calculate the Jaccard containment. This allows the rapid evaluation of the relationship between a pair of multiple sequence alignments. That is, does one alignment contain a superset or subset of the regions in the other.

This method is intended to enable an automated quality control for protein family profile HMMs. The MinHash derived comparison method for protein families is a critical component of an automated pipeline for identifying families from sequence similarity search which are candidates for integration with Pfam. This method can be adjusted to meet the required speed for an interactive protein sequence similarity search by slightly reducing the accuracy of the estimate.

We foresee multiple applications for these methods. They could be used to filter user submitted family profile HMMs, enabling crowdsourcing of the Pfam database. We also see the hash-based set relationship comparison methods as useful not just for protein families, but for other types of sequence data, such as RNA families. In addition, the calculation of Jaccard containment can be used in the hierarchical classifications such as InterPro or SUPERFAMILY to help identify subfamily relationship ^13, 14^.

## Data availability

An implementation of the method discussed in this paper is available in the Search-Sifter package. DOI: http://doi.org/10.5281/zenodo.1560659 ^15^.

## Software availability

**The Search-Sifter package is available at:** https://github.com/bateman-research/search-sifter.

**Archived source code at time of publication:** http://doi.org/10.5281/zenodo.1560659 ^15^.

**License:** MIT License.
