# Supplementary material for: Rapid identification of novel protein families using similarity searches
Source: F1000Res. 2018 Dec 24;7:ISCB Comm J-1975. [Version 1] doi: 10.12688/f1000research.17315.1 (PMC6439793; doi:10.12688/f1000research.17315.1)
Supplement: Peer review history

# Peer review: Reviewer response for version 1

**DOI:** 10.5256/f1000research.18934.r46291

**Published:** 2019-03-28

**Authors:** Desmond G Higgins

**Affiliations:** 1 School of Biomedical and Biomolecular Science and UCD Conway Institute of Biomolecular and Biomedical Research, Conway Institute, University College Dublin, Dublin, Ireland

This paper describes a method for very quickly comparing lists of sequence parts (e.g. from a similarity search) to PFAM families. It is extremely well described. I have a few typos and queries but these are very minor. The method looks useful and, as the authors point out, can be used as the basis for a variety of production or interactive tools.

 I have no major changes to request.

 Minor points:

1. page 3. The word "regions" is mentioned twice. What is a region?
2. page 4 "But in the other 2 cases we may also wish to". Wish to what? Also "We can calculate theb"
3. page 5. The minimum of the three is used. Minimum time?
4. "chunking"? Is this a word?
5. page 3 "This overlap criterION is an important ..."
6. page 6 "...identifying families from A sequence similarity search."

I have read this submission. I believe that I have an appropriate level of expertise to confirm that it is of an acceptable scientific standard.

# Peer review: Reviewer response for version 1

**DOI:** 10.5256/f1000research.18934.r44111

**Published:** 2019-02-11

**Authors:** Daniel J. Rigden

**Affiliations:** 1 Institute of Integrative Biology, University of Liverpool, Liverpool, UK

This concise paper nicely describes a problem - how to rapidly compare the results of a database search to a set of protein families - and provides a solution via an efficient hashing algorithm. I have just a few suggestions.

- To me a title like "Rapid identification of novel protein families in the results of similarity searches" would better convey the content. As is, it reads as if the paper is more about innovations in the similarity searches themselves.
- The authors should cite UniProtKB in the Intro.
- Typo 'theb Jaccard containment'.
- Maybe a brief explanation of what hashing is - the paper rather assumes the reader knows what it means.
- Are the funny partial square brackets round i/w on p.5 correct?
- Maybe 'In Figure 4, the time taken for calculation of the...'
- Maybe on pg. 5 spell out what the consequences of under- or over-prediction of the two Jaccard measurements would be for placing database search results into the three categories defined at the star of Methods.
- On p. 5 the authors say that 'Increasing the value of w also reduces accuracy' Visually, that seems clear for n=64 but the differences for n=1, 4 or 16 are much less obvious. Maybe calculate Rsquared or some other statistical measure to quantify this tendency.

I have read this submission. I believe that I have an appropriate level of expertise to confirm that it is of an acceptable scientific standard.
